# Thermodynamic and structural contributions of the 6-thioguanosine residue to helical properties of RNA

**DOI:** 10.1038/s41598-019-40715-2

**Published:** 2019-03-13

**Authors:** Michał Gładysz, Witold Andrałojć, Tomasz Czapik, Zofia Gdaniec, Ryszard Kierzek

**Affiliations:** Institute of Bioorganic Chemistry, Polish Academy of Sciences, 61-704 Poznan, Noskowskiego 12/14, Poland

## Abstract

Thionucleotides, especially 4-thiouridine and 6-thioguanosine, are photosensitive molecules that photocrosslink to both proteins and nucleic acids, and this feature is a major reason for their application in various investigations. To get insight into the thermodynamic and structural contributions of 6-thioguanosine to the properties of RNA duplexes a systematic study was performed. In a series of RNA duplexes, selected guanosine residues located in G-C base pairs, mismatches (G-G, G-U, and G-A), or 5′ and 3′-dangling ends were replaced with 6-thioguanosine. Generally, the presence of 6-thioguanosine diminishes the thermodynamic stability of RNA duplexes. This effect depends on its position within duplexes and the sequence of adjacent base pairs. However, when placed at a dangling end a 6-thioguanosine residue actually exerts a weak stabilizing effect. Furthermore, the structural effect of 6-thioguanosine substitution appears to be minimal based on NMR and Circular Dichroism (CD) data.

## Introduction

RNA contains many modified nucleotides performing various biological functions, and thionucleotides constitute one group of these modified nucleotides. The following thionucleotides can be found among the 112 entries of the RNA modification database (http://mods.rna.albany.edu): 2-thiocytidine, 2-thiouridine, 4-thiouridine, 5-methyl-2-thiouridine, 2-thio-2′-O-methyluridine, 5-methoxycarbonylmethyl-2-thiouridine, 5-aminomethyl-2-thiouridine, 5-methylaminomethyl-2-thiouridine, 5-(isopentenyl aminomethyl)-2-thiouridine, geranylated 5-methylaminomethyl-2-thiouridine and geranylated 5-carboxymethylaminomethyl-2-thiouridine. In addition to the thionucleotides listed above, there are also five 2-methylthio-N6-substituted derivatives of adenosine. The biological functions of some of these thionucleotides are discussed in several reviews^1,2^.

In addition to naturally occurring thionucleotides, various synthetic analogues have been devised to achieve specific goals. One family of these analogues is constituted by 6-thioguanosine derivatives. Thionucleotides, especially 4-thiouridine (s4U) and 6-thioguanosine (s6G), are photosensitive molecules that photocrosslink to both proteins and nucleic acids, and this feature is a major reason for their application in various investigations^3–10^. In the context of duplex RNA, it has been shown that 4-thiouridine efficiently crosslinks to nucleotides in the opposite strand^7^. Photophysics and photochemistry of these crosslinking processes on the level of nucleosides and oligonucleotides is very well documented^6,9^. The application of 6-thioguanosine to photocrosslinking is somewhat less established. Nevertheless, there are several reports on inter- and intramolecular photocrosslinking of RNAs containing 6-thioguanosine^6,11,12^. Thionucleotides, particularly 6-thioguanosine, undergo photo induced oxidation. Aside from inter- and intramolecular photocrosslinking of RNA, photocrosslinking of RNAs and proteins has also been reported^6,12,13^. 6-Thioguanosine was found to be a useful tool for structural and functional studies of RNAs including activity of ribonuclease P^14^, interaction of cap analogs^15^, triplexes formation^16^ and RNA sequencing and population dynamics^17,18^.

It is important to evaluate whether the substitution of uridine with 4-thiouridine and guanosine with 6-thioguanosine influences the structure and stability of RNA. Studies of RNA duplex formation by oligonucleotides with either an internal or a terminal 4-thiouridine showed an increase in their thermodynamic stability and indicated that the presence of s4U increases the stability of base pairing with guanosine more than that with adenosine^19^. In contrast, for the same set of oligonucleotides modified with 2-thiouridine (s2U), it has been shown that s2U increases the stability of base pairing with adenosine more than that with guanosine. This correlation indicated that s2U preferentially pairs with adenosine over guanosine. To the best of our knowledge, there are no extensive studies concerning the contribution of s6G to the thermodynamic stabilities of RNA duplexes. However, it was previously reported that the incorporation of 2′-deoxy-6-thioguanosine diminishes the thermodynamic stability of DNA duplexes^20^. Extending these studies to RNA duplexes is important because there are several reports on the application of 6-thioguanosine residues for inter- and intramolecular photocrosslinking of RNAs and photocrosslinking of RNAs and proteins^6,11,12^. For example, derivatives of 6-thioguanosine have been incorporated into RNA via transcription with the 5′-O-triphosphate of 6-thioguanosine (s6GTP)^21^. A mixture of GTP and s6GTP was used for transcription, which resulted in the incorporation of a single s6G into target RNA. Incorporation of a single s6G into RNA did not change the overall structure of large RNAs; however, a local structural rearrangement is possible.

In this report, we performed extensive studies to obtain detailed information on the influence of s6G on the thermodynamic stabilities of RNA duplexes and selected guanosines located in G-C base pairs, mismatches (G-G, G-U, and G-A), and 5′ and 3′-dangling ends were replaced with 6-thioguanosine. Together, eleven 6-thioguanosine containing RNA duplexes were investigated. The influence of s6G on the thermodynamic stability of RNA was obtained by comparison with unmodified duplexes with guanosine at the same position. Generally, the presence of 6-thioguanosine diminished the thermodynamic stability of RNA duplexes, however, this effect depended on its position within duplexes and the sequence of adjacent base pairs. Furthermore, the structural effect of 6-thioguanosine substitution appeared to be minimal based on NMR and Circular Dichroism (CD) data.

## Materials and Methods

### Materials

Guanosine that was used for synthesis and most other reagents (acetic anhydride, isobutyryl chloride, 2,4-dinitro-1-fluorobenzen, tert-butyldimethylsilyl chloride and Lawesson’s reagent) were purchased from Sigma-Aldrich. Dimethoxytrityl chloride and 2-cyanoethyl N,N,N′,N′-tetraisopropylphosphorodiamidite were purchased from ChemGenes. Solvents were used at the highest available purity and dried with molecular sieves, if necessary. The chemical stepwise synthesis of 3′-O-phosphoramidite of the protected 6-thioguanosine is described in detail in Supplementary Materials.

### Oligonucleotide synthesis

Oligonucleotides were synthesized on a BioAutomation MerMade12 DNA/RNA synthesizer using β-cyanoethyl phosphoramidite chemistry^22^ and commercially available phosphoramidites (ChemGenes, GenePharma). The synthesis was conducted using a standard RNA protocol, except that milder conditions were used for oxidation (0.02 M iodine in pyridine/water, 95/5 v/v). For deprotection, 6-thioguanosine oligoribonucleotides were treated with a mixture of isoamylamine/pyridine (2/1 v/v) for 24 hours at room temperature^23,24^. Silyl-protecting groups were cleaved by treatment with triethylamine  trihydrofluoride. Deprotected oligonucleotides were purified by silica gel thin layer chromatography (TLC) in 1-propanol/aqueous ammonia/water (55/35/10 v/v/v), as described in detail previously^23^.

### UV melting experiments

Thermodynamic measurements were performed for nine different concentrations of RNA duplex in the range of 10^−3^–10^−6^ M on a JASCO V-650 UV/Vis spectrophotometer in buffer containing 1 M sodium chloride, 20 mM sodium cacodylate, and 0.5 mM Na_2_EDTA (pH 7)^25^. Oligonucleotide single strand concentrations were calculated from the absorbance above 80 °C, and single strand extinction coefficients were approximated by a nearest-neighbor model. The extinction coefficient of 6-thioguanosine was considered the same as for guanosine^26^. Absorbance vs. temperature melting curves were measured at a wavelength of 260 nm with a heating rate of 1 °C/min from 0 to 90 °C on a JASCO V-650 spectrophotometer with a thermoprogrammer. The melting curves were analyzed, and the thermodynamic parameters were calculated from a two-state model with MeltWin 3.5 software^27^. For most sequences, ∆H° derived from T_M_^−1^ vs. ln(C_T_/4) plots was within 15% of that derived from averaging the fits to individual melting curves, which was expected if the two-state model is reasonable.

### CD spectra

CD spectra were recorded on a JASCO J-815 spectropolarimeter using 1.5 mL quartz cuvettes with a 5 mm path length. Oligonucleotides were dissolved in a buffer containing 1 M sodium chloride, 20 mM sodium cacodylate and 0.5 mM Na_2_EDTA (pH 7.0) to achieve a 20 µM sample concentration. All samples were denatured for 3 min at 90 °C and then slowly cooled to room temperature overnight before data collection. The measurements were taken at 5 °C in the 205–350 nm wavelength range with a 1 nm data point interval. CD curves were established as an average of three CD measurements. The buffer spectrum was subtracted from the sample spectra.

### NMR spectroscopy of RNA duplexes

For the acquisition of NMR data, all RNA duplexes were dissolved in a 10 mM sodium phosphate buffer (pH 6.8) containing 150 mM sodium chloride and 0.1 mM Na_2_EDTA. To ensure that the RNA was uniquely present in the duplex form, samples were further washed with the same buffer on an Amicon centrifugal filter with a 3 kDa molecular weight cutoff (any excess single-stranded RNA would pass through the pores of the Amicon membrane). NMR data were collected on a Bruker AVANCE III 700 MHz spectrometer, equipped with a QCI CryoProbe. 1D proton NMR spectra as well as 2D ^1^H-^1^H NOESY spectra were measured in H_2_O/D_2_O (9/1, v/v) at 25 °C and 5 °C. An additional set of NOESY spectra was acquired in 99.990% D_2_O (Sigma Aldrich) at 25 °C to facilitate the assignment of nonexchangeable protons. All the observable imino and amino protons as well as the nonexchangeable aromatic and anomeric protons were assigned using standard methodologies^28^, as described in the Supplementary Materials and exemplified in Figs S1–S3.

## Results and Discussion

### Chemical synthesis of the protected derivative of 3′-O-phosphoramidite 6-thioguanosine and oligoribonucleotides containing a 6-thioguanosine residue

Several approaches for the synthesis of protected 6-thioguanosine are described in the literature, and the major difference between them concerns the S-protecting group used during synthesis. 2,4-Dinitrophenyl^29^, 2-cyanoethyl^30^ and 2-[(2-ethylhexyl) oxycarbonyl] ethyl^31^ were all applied previously for the protection of the sulfur atom; in this study, the 2,4-dinitrophenyl group was used (Scheme S1). Guanosine was converted with acetic anhydride into 5′,3′,2′-O-triacetylguanosine, followed by a reaction with isobutyl chloride to obtain the N4-isobutyryl derivative. Acyl-protected guanosine was converted into a 6-thioderivative by treatment with Lawesson’s reagent. Treatment under mild conditions with a methanol and aqueous ammonia mixture resulted in the formation of N2-isobutyryl-6-thioguanosine. This derivative was converted into 5′-O-dimethoxytrityl, followed by treatment with 1-fluoro-2,4-dinitrobenzene to obtain 5′-O-dimethoxytrityl-N2-isobutyryl-6S-(2,4-dinitrophenyl) guanosine. A subsequent reaction with t-butyldimethylsilyl chloride resulted in the formation of a mixture of 2′- and 3′-silyl isomers. After separation, the 2′-O-silyl derivative was converted into 3′-O-phosphoramidite by treatment with 2-cyanoethyl N,N,N′,N′-tetraisopropylphosphorodiamidite^29,32^.

### Thermodynamics of duplexes containing 6-thioguanosine

Two sets of RNA duplexes were selected as templates for our study. In both of these sets, either guanosine or s6G residues were placed, one residue at a time, in various sequential contexts, including the internal and terminal base pairs within the duplex as well as 5′- or 3′-dangling ends (Table 1). The internal position of the s6G residue was also involved in mismatches with U, G and A.

**Table 1:** Thermodynamic parameters of duplex formation with s6G.

	Duplexes (5′-3′)^b^	Average of curve fits				T_M_^−1^ vs log C_T_ plots				
		−ΔH° (kcal/mol)	−ΔS° (eu)	−ΔG°_37_ (kcal/mol)	T_M_^c^ (°C)	−ΔH° (kcal/mol)	−ΔS° (eu)	−ΔG°_37_ (kcal/mol)	T_M_^c^(°C)	ΔΔG°_37_ (kcal/mol)^d^
C1	CAGUCAGU GUCAGUCA	71.0 ± 3.5	196.6 ± 11.0	10.04 ± 0.14	53.1	77.6 ± 4.0	217.1 ± 12.4	10.26 ± 0.15	52.7	—
C2	UCAGUCAG AGUCAGUC	73.5 ± 5.0	204.5 ± 15.6	10.07 ± 0.22	52.7	71.2 ± 3.9	197.4 ± 12.3	9.96 ± 0.15	52.7	—
M1	**G**CAGUCAGU GUCAGUCA	74.3 ± 1.0	204.5 ± 3.0	10.89 ± 0.11	56.3	63.6 ± 1.6	171.6 ± 5.0	10.41 ± 0.07	57.1	0
M2	***G***CAGUCAGU GUCAGUCA	75.3 ± 11.5	207.5 ± 35.3	10.95 ± 0.57	56.3	74.4 ± 3.7	204.7 ± 11.4	10.96 ± 0.18	56.6	−0.55
M3	UCAGUCAG**G** AGUCAGUC	72.0 ± 6.8	196.8 ± 20.9	10.99 ± 0.30	57.5	72.9 ± 3.8	199.5 ± 11.7	10.98 ± 0.19	57.2	0
M4	UCAGUCAG***G*** AGUCAGUC	87.5 ± 19.7	244.6 ± 60.4	11.61 ± 0.98	56.1	87.7 ± 11.6	245.1 ± 35.4	11.70 ± 0.66	56.4	−0.72
M5	**G**CAGUCAGU **C**GUCAGUCA	83.3 ± 7.8	225.1 ± 23.0	13.48 ± 0.66	65.2	74.2 ± 2.5	198.2 ± 7.4	12.74 ± 0.19	65.3	0
M6	***G***CAGUCAGU **C**GUCAGUCA	78.4 ± 1.0	213.6 ± 3.2	12.18 ± 0.09	61.1	69.6 ± 2.5	186.9 ± 7.6	11.67 ± 0.15	61.7	1.07
M7	UCAGUCAG**G** AGUCAGUC**C**	76.8 ± 6.9	206.4 ± 20.8	12.79 ± 0.44	64.5	73.3 ± 2.4	195.8 ± 19.3	12.53 ± 0.47	64.7	0
M8	UCAGUCAG***G*** AGUCAGUC**C**	77.6 ± 2.7	211.3 ± 8.1	12.03 ± 0.23	60.7	67.8 ± 1.6	181.6 ± 5.0	11.44 ± 0.10	61.2	1.09
M9	UCAG**G**CAGU AGUC**C**GUCA	80.2 ± 2.0	213.0 ± 5.9	13.93 ± 0.17	68.6	77.6 ± 0.7	206.0 ± 2.1	13.74 ± 0.06	68.7	0
M10	UCAG***G***CAGU AGUC**C**GUCA	68.4 ± 2.1	184.5 ± 6.8	11.17 ± 0.09	59.6	66.9 ± 2.8	180.1 ± 8.6	11.10 ± 0.16	59.7	2.64
M11	UCAC**G**GAGU AGUG**C**CUCA	78.9 ± 8.7	210.4 ± 25.8	13.69 ± 0.75	67.9	75.7 ± 2.9	201.0 ± 8.6	13.37 ± 0.23	67.8	0
M12	UCAC***G***GAGU AGUG**C**CUCA	70.0 ± 8.8	189.7 ± 27.0	11.19 ± 0.44	59.1	63.9 ± 3.8	170.9 ± 11.7	10.89 ± 0.22	59.7	2.48
M13	UCAA**G**UAGU AGUU**C**AUCA	75.6 ± 6.3	210.5 ± 19.7	10.34 ± 0.18	53.5	73.8 ± 3.6	204.8 ± 11.2	10.28 ± 0.15	53.6	0
M14	UCAA***G***UAGU AGUU**C**AUCA	69.4 ± 2.0	195.8 ± 6.4	8.67 ± 0.07	46.9	62.4 ± 2.7	173.8 ± 8.4	8.53 ± 0.06	47.2	1.75
M15	UCAU**G**AAGU AGUA**C**UUCA	76.0 ± 9.6	212.6 ± 30.1	10.12 ± 0.27	52.3	78.6 ± 1.3	220.6 ± 4.0	10.19 ± 0.05	52.1	0
M16	UCAU***G***AAGU AGUA**C**UUCA	61.0 ± 3.8	172.3 ± 12.0	7.59 ± 0.11	42.5	62.2 ± 2.0	176.2 ± 6.3	7.61 ± 0.02	42.5	2.58
M17	UCAG**G**CAGU AGUC**G**GUCA	77.3 ± 1.9	217.4 ± 5.8	9.92 ± 0.11	51.2	85.0 ± 2.5	240.9 ± 7.7	10.30 ± 0.12	51.4	0
M18	UCAG***G***CAGU AGUC**G**GUCA	62.3 ± 2.5	175.7 ± 7.9	7.76 ± 0.10	43.2	62.1 ± 2.6	175.1 ± 8.4	7.74 ± 0.04	43.2	2.56
M19	UCAG**G**CAGU AGUC**U**GUCA	83.5 ± 3.4	230.6 ± 10.4	12.03 ± 0.17	58.9	74.2 ± 1.4	202.0 ± 4.4	11.53 ± 0.08	59.4	0
M20	UCAG***G***CAGU AGUC**U**GUCA	73.1 ± 2.4	204.9 ± 7.6	9.55 ± 0.07	50.4	73.8 ± 1.4	207.2 ± 4.5	9.56 ± 0.04	50.3	1.97
M21	UCAG**G**CAGU AGUC**A**GUCA	68.7 ± 8.3	194.2 ± 26.5	8.49 ± 0.15	46.1	69.3 ± 3.7	196.3 ± 11.8	8.45 ± 0.08	45.9	0
M22	UCAG***G***CAGU AGUC**A**GUCA	57.8 ± 2.1	164.8 ± 6.8	6.66 ± 0.06	37.7	58.5 ± 2.3	167.4 ± 7.4	6.63 ± 0.03	37.5	1.82

^a^solutions: 1 M sodium chloride, 20 mM sodium cacodylate, 0.5 mM Na_2_EDTA, pH 7.0, ^b^***G*** – 6-thioguanosine, ^c^calculated for 10^−4^ M oligomer concentration, ^d^between the duplexes of the same sequence containing ***G*** vs ***G***.

### 6-Thioguanosine at the internal position

The effect of a nucleotide modification depends on its position within an RNA duplex. Modifications that contribute to stabilization and destabilization of duplexes are the most effective when placed in the center of an RNA duplex^25,33–35^. Moreover, the magnitude of this stabilizing or destabilizing effect is dependent on the sequence and arrangement of 5′- and 3′-adjacent base pairs. In this study, s6G-C pairs were placed in four arrangements of A-U and G-C in 5′- and 3′-adjacent base pairs (see duplexes M10, M12, M14 and M16 in Table 1).

We found that replacing G with s6G in a base pair with C always destabilized RNA duplexes and the effectiveness of the destabilization was dependent on the sequence and arrangement of adjacent base pairs. The thermodynamic stability (−ΔG°_37_) of the reference duplexes M9, M11, M13 and M15 were 13.74, 13.37, 10.28 and 10.19 kcal/mol, respectively. The replacement of guanosine with 6-thioguanosine at the central position in duplexes M10, M12, M14 and M16 significantly diminished their thermodynamic stability to 11.10, 10.89, 8.53 and 7.61 kcal/mol, respectively. The changes in the thermodynamic stability (ΔΔG°_37_) of duplexes for 5′UGA/3′ACU, 5′AGU/3′UCA, 5′CGG/3′GCC, and 5′GGC/3′CCG (where G corresponds to 6-thioguanosine) 6-thioguanosine arrangements were 2.58, 1.75, 2.48 and 2.64 kcal/mol, respectively. Thus, for three among the tested arrangements, the destabilizing effect of 6-thioguanosine was consistently around 2.5–2.6 kcal/mol, while for the fourth one, containing an adenosine residue 5′-adjacent to 6-thioguanosine, the destabilizing effect was around 30% weaker.

### 6-thioguanosine at terminal positions

6-Thioguanosine was also introduced in 5′- and 3′-terminal base pairs. Replacement of G with s6G at these terminal base pairs in duplex pairs M5-M6 and M7-M8 resulted in the destabilization of RNA duplexes by 1.07 and 1.09 kcal/mol, respectively.

In general, the presence of 5′- and 3′-dangling ends stabilizes RNA duplexes^36–38^. This effect is particularly strong for 3′-terminal dangling ends, and the stabilization effect depends on the identity of both the dangling end and the 5′-adjacent base pair. In this study, the addition of 5′- or 3′- unpaired guanosine to the reference C1 and C2 duplexes resulted in the formation of M1 and M3 duplexes with thermodynamic stabilities (−ΔG°_37_) of 10.41 and 10.98 kcal/mol, respectively. This finding indicates that due to the presence of 5′- or 3′-dangling ends, the stability of M1 and M3 duplexes increased by 0.15 and 1.02 kcal/mol, respectively. Alternatively, placing 6-thioguanosine at the 5′- and 3′-dangling ends led to the formation of M2 and M4 duplexes with thermodynamic stabilities of 10.96 and 11.70 kcal/mol, respectively (the stability of M2 and M4 duplexes increased by 0.70 and 1.74 kcal/mol relative to C1 and C2, respectively). Therefore, it can be concluded that replacing guanosine with 6-thioguanosine contributes to the stability of RNA duplexes by 0.55 and 0.72 kcal/mol for 5′- and 3′-dangling ends, respectively.

It is postulated that the thermodynamic effects of dangling ends are related to their stacking ability^37,38^. Stacking interactions of a terminal base pair (considered as 5′- and 3′-dangling ends) and hydrogen bonding of terminal base pairs both contribute to the thermodynamic stability of a terminal base pair. Comparisons of the stabilization effects of 6-thioguanosine on 5′- and 3′-dangling ends with the destabilization effect of a terminal s6G-C base pair suggest that weakened hydrogen bonding is responsible for the overall destabilization effect of the terminal s6G-C base pair^39^. A compensation between the effects of stacking and hydrogen bonding is very often observed in RNA^39^.

### 6-Thioguanosine forms mismatches with G, U or A in RNA duplexes

Guanosine is a unique nucleotide that, in addition to its canonical interactions with C, also pairs with A, G and U^40–43^. In comparison to canonical G-C base pair mismatches, G-G, G-A and G-U stabilize RNA duplexes much less efficiently. Indeed, the thermodynamic stability of duplex M9 with a canonical G-C base pair and duplexes with G-G (M17), G-U (M19) and G-A (M21) mismatches were 13.74, 10.30, 11.53 and 8.45 kcal/mol, respectively. These results show that the presence of G-G, G-U and G-A mismatches destabilizes the canonical duplex M9 by 3.44, 2.21 and 5.29 kcal/mol, respectively. Replacing guanosine with 6-thioguanosine results in the formation of either the fully complementary duplex (M10) or duplexes containing s6G-G (M18), s6G-U (M20) and s6G-A (M22) mismatches with thermodynamic stabilities of 11.10, 7.74, 9.56 and 6.63 kcal/mol, respectively. These results indicate that the presence s6G in duplexes containing s6G-G (M18), s6G-U (M20) and s6G-A (M22) mismatches diminished destabilization by 3.36, 1.54 and 4.27 kcal/mol, respectively. A comparison of the destabilization effect resulting from the presence of mismatches with guanosine and 6-thioguanosine indicated that the mismatches formed by s6G were further destabilized (ΔΔG°_37_) by 0.29, 0.30 and 0.65 kcal/mol when s6G formed mismatches with G, U and A, respectively.

### Circular dichroism investigations

Circular dichroism spectra were recorded at 5 °C in the same buffer as that used in UV melting experiments and at similar concentrations of RNA duplexes. All studied RNA duplexes showed CD spectra typical of A-RNA double stranded helices^44^. The replacement of guanosine with 6-thioguanosine within the tested RNA duplexes containing base-paired and mismatched s6G results in red shift of the maximum ca. 260 nm by 5–10 nm, without altering the overall shape of the spectrum (Fig. S1a–d,f,g). Only for duplex M18, which contains s6G-G mismatch, no shift of maximum was observed (Fig. S1e). For most 6-thioguanosine-containing RNA duplexes, the CD spectra contained a strong positive peak in the range of 260–270 nm and a weak positive peak at approximately 225 nm. Strong negative peaks were observed at approximately 210–215 nm, and weak negative peaks were observed at 240 and 295 nm.

**Figure 1:**
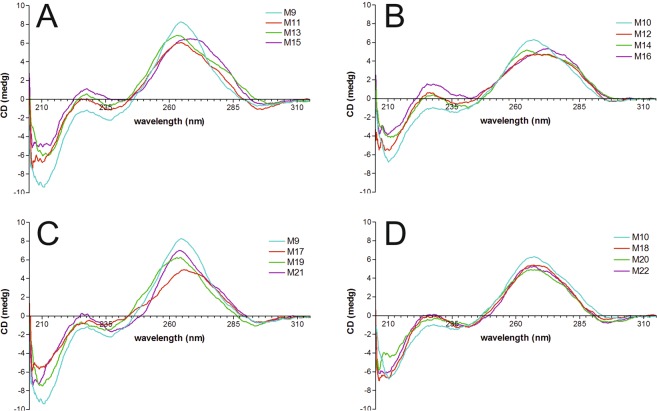
The CD spectra of the duplexes containing: (**A**) the G-C base pair in different sequential contexts (duplexes M9, M11, M13 and M15), (**B**) the s6G-C base pair in different sequential contexts (duplexes M10, M12, M14 and M16), (**C**) all the G-X base pairs/mismatches in a single sequential context (duplexes M9, M17, M19 and M21) and (**D**) all the s6G-X base pairs/mismatches in a single sequential context (duplexes M10, M18, M20 and M22). X is A, G or U.

Figure 1A shows the CD spectra of M9, M11, M13 and M15 RNA duplexes containing a G-C base pair at the center surrounded by different 5′- and 3′-adjacent base pairs. CD spectra of M9, M11 and M13 duplexes were comparable to each other; for M15 only, the positive peak shifted by 6–7 nm to 272 nm. For the corresponding 6-thioguanosine-modified duplexes (M10, M12, M14 and M16), the strong positive peaks shifted by 5–7 nm (Fig. 1B). The maxima of the positive peaks were situated at 267, 270, 262 and 272 nm for M10, M12, M14 and M16 duplexes, respectively. Moreover, peaks for M12, M14 and M16 were broader than the peak for M10.

The influence of the replacement of guanosine with 6-thioguanosine within G-G, G-U and G-A mismatches was also studied. CD spectra containing G-G, G-U and G-A mismatches (Fig. 1C) and the modified s6G-G, s6G-U and s6G-A mismatches (Fig. 1D) were comparable.

### NMR analysis of s6G-containing RNA duplexes

**Figure 2:**
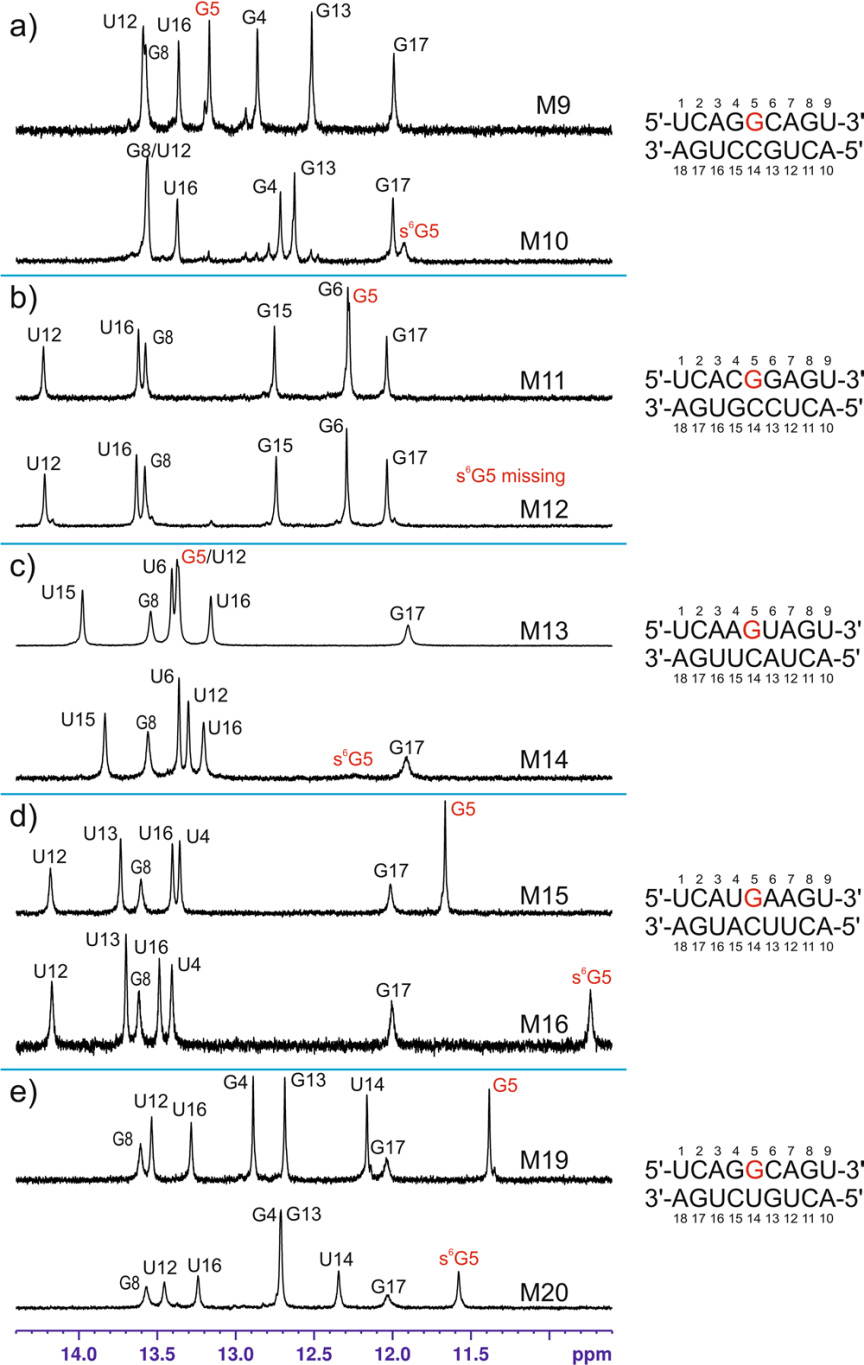
The effect of the G to s6G substitution on the imino region of the NMR spectra for (**a**) the M9-M10 duplex pair, (**b**) the M11-M12 duplex pair, (**c**) the M13-M14 duplex pair, (**d**) the M15-M16 duplex pair and (**e**) the M19-M20 duplex pair.

To more thoroughly evaluate the structural consequences of the G to s6G substitution, four RNA duplexes containing s6G-C pairs at internal positions (M10, M12, M14 and M16), as well as their unmodified counterparts (M9, M11, M13 and M15), were investigated by NMR spectroscopy. The chemical shift changes (ΔCS) induced by the G to s6G modification were rather limited and qualitatively very similar for all four studied pairs of duplexes. The ΔCS observed for the M9-M10 and M11-M12 pairs of duplexes are shown in Tables S1 and S2, respectively. Among the assigned nonexchangeable protons, no ΔCS greater than 0.15 ppm was observed. Moreover, ΔCS > 0.05 ppm were observed exclusively for atoms belonging to the internal G-C base pair (pair bearing the s6G modification) and the two base pairs directly flanking it. The ΔCS experienced by exchangeable protons were greater (Tables S1 and S2) but still localized in the direct vicinity of the modified base pair. Overall, these observations strongly suggest that the structural rearrangements induced by s6G were minor and only limited to the closest neighboring base pairs of the modified base pairs. Similar conclusions were previously obtained for DNA duplexes modified by s6dG^45,46^. Figure 2a–d shows the assigned imino proton region of the ^1^H-NMR spectra of the studied duplexes. Most spectra feature seven imino resonances, lacking only those belonging to the terminal A-U base pairs. A feature that was recurrent for all four pairs of duplexes was a considerable broadening of the G5 imino proton upon the introduction of the thio-modification, accompanied by a ca. 1 ppm upfield shift of this resonance (in the range of 0.93–1.27 ppm for the different duplexes). The line broadening was severe enough that for M14, the G5 imino proton was barely visible at 25 °C, while for M12, it only became observable at 5 °C (Fig. S4). This broadening can be interpreted as an indicator of an increased rate of exchange of the imino proton with the solvent. These observations indicate that the s6G modification weakens the G-C base pairing interactions in accordance with our thermodynamic results, yet does not abolish them completely. Another recurring spectral feature for all studied duplexes was a consistent ca. 0.25–0.4 ppm downfield shift of the cytidine amino proton directly hydrogen bonding to the sulfur (S6) atom (in the range of 0.24–0.42 ppm for the different duplexes). A similar pattern of ΔCS, a ca. 1 ppm *upfield* shift of the G imino proton and a ca. 0.25 ppm *downfield* shift of the C amino proton, was previously reported for a s6dG-dC base pair in a DNA duplex^46^. With our current observation of this pattern in four different RNA duplexes, we can conclude that this pattern is a general NMR spectral ‘fingerprint’ of an s6G-C base pair in both RNA and DNA.

An additional pair of duplexes, containing either a G-U wobble base pair (M19) or its s6G-modified counterpart (M20) at an internal position, was also investigated by NMR. The ΔCS measured for nonexchangeable protons were comparable to those observed for the duplexes with modified G-C pairs and, once again, localized in direct proximity of the modified base pair. This finding suggests that also for the G-U base pair, the G to s6G substitution results in very minimal structural rearrangements. The imino protons of G and U residues involved in the s6G-U wobble pair were both shifted by ca. 0.2 ppm *downfield* with respect to the nonmodified duplex (Fig. 2e). In contrast with the results obtained for the s6G-C pair, these resonances were not appreciably broadened by exchange at room temperature. However, when the temperature was increased, it became apparent that the s6G modification considerably weakened the wobble base pair. For the modified duplex M20, the relevant imino proton peaks became noticeably broadened at 35 °C and disappeared completely from the spectra at ca. 45 °C, while for the nonmodified M19, these peaks remained narrow at ca. 45 °C (Fig. S5). This NMR observation concurs with our thermodynamic results indicating that the s6G-U-containing duplex M20 is significantly destabilized with respect to the unmodified M19.

Interestingly, contrary results were previously observed for DNA. Both NMR^46^ and thermodynamic studies^47^ indicated that, for a s6dG-T wobble pair in a DNA duplex, the s6G modification actually slightly stabilized the structure. In order to formulate a plausible explanation for this difference, the effects of s6G introduction into G-C pairs in DNA and RNA were also compared. The DNA study by Bohon and de los Santos^47^ reports an approximately 0.9 kcal/mol destabilization at 37 °C accompanied by around 3 °C decrease in T_M_ when s6G is introduced in the 5′T**G**A/3′ACT sequential context. On the other hand, our current data reveal a 2.6 kcal/mol destabilization and around 10 °C drop in T_M_ when the same substitution is made in RNA. Thus, the destabilizing effect observed in RNA appears to be much more pronounced. Taken together, the results for s6G-C base pairs and for the s6G-U mismatches could indicate that the s6G modification is easier to accommodate in B-form helices (DNA) compared to A-form helices (RNA). However, much caution is required when directly comparing these data due to different polymer types and different overall sequences.

## Conclusions

6-Thioguanosine residues were placed in duplexes at central and terminal positions with various sequences and arrangements of 5′- and 3′-adjacent base pairs, as well as, at 3′- and 5′-dangling ends. Placing the s6G residue at the center of RNA duplexes diminished their thermodynamic stability up to 2.64 kcal/mol, and this destabilizing effect did not substantially depend on the sequence and arrangement of 5′- and 3′-adjacent pairs. The presence of 6-thioguanosine in 5- or 3′-terminal base pairs of the duplexes reduced their stability by ca. 1 kcal/mol. However, when guanosine 5′- and 3′-dangling ends were replaced with the s6G residue, the thermodynamic stability of RNA duplexes was enhanced by 0.5–0.7 kcal/mol. Comparisons of the influence of s6G involved in base pair formation as well as its presence on 5′- and 3′-dangling ends suggest that replacing guanosine with s6G led to increased stacking interactions, while diminishing hydrogen bonding strength within RNA duplexes.

The presence of the s6G modification within s6G-G, s6G-U and s6G-A mismatches also destabilized RNA duplexes; however, this effect was smaller than the destabilization due to the introduction of G-G, G-U and G-A mismatches themselves without s6G modification.

NMR investigations indicated that the structures of RNA duplexes containing natural base pairs and base pairs with s6G modification were similar. This observation is consistent with the thermodynamic data and CD spectra reported herein, as well as, with the previously reported results for DNA duplexes containing 2′-deoxy-6-thioguanosine. Taken together, our results demonstrate that the presence of 6-thioguanosine in helical regions of RNA only negligibly affects their structure. This finding is reassuring for photocrosslinking studies in which a single 6-thioguanosine residue is usually present in the group of RNAs as its presence should not alter the formed secondary structures.

On the other hand, in some special cases minor structural and electronic changes induced by the 6-thioguanosine substitution may significantly affect the properties of RNA molecules. For example, a 6-thioguanosine introduced at the active sites of ribozymes may be enough to affect their activity. Thus, in this context, a G to s6G substitution could provide valuable data regarding the mechanisms of action of these types of RNA molecules^14,48^.

## Supplementary information

Supplementary Info
