# Supplementary material for: Thermodynamic and structural contributions of the 6-thioguanosine residue to helical properties of RNA
Source: Sci Rep. 2019 Mar 13;9:4385. doi: 10.1038/s41598-019-40715-2 (PMC6416399; doi:10.1038/s41598-019-40715-2)
Supplement: Supplementary file 1 — Supplementary Info [file 41598_2019_40715_MOESM1_ESM.docx]

**Supplementary Material**

**Thermodynamic and structural contributions of the 6-thioguanosine residue to helical properties of RNA**

Michał Gładysz^†^, Witold Andrałojć^†^, Tomasz Czapik, Zofia Gdaniec and Ryszard Kierzek*

Institute of Bioorganic Chemistry, Polish Academy of Sciences, 61-704 Poznan, Noskowskiego 12/14, Poland

^†^The first two authors should be regarded as joint first authors

* To whom correspondence should be addressed: Tel: +48 61 8528503; Fax: +48 61 8520532; Email: [rkierzek@ibch.poznan.pl](mailto:rkierzek@ibch.poznan.pl)

Contents:

Supplementary methods

Schematic S1 Chemical synthesis of protected 6-thioguanosine amidite

Figure S1 The CD spectra recorded for the duplex pairs.

Figure S2. The aromatic-to-anomeric section of the NOESY spectra recorded at 25 °C in D2O for the duplexes M9 and M10.

Figure S3. The imino-imino and imino-amino sections of the NOESY spectra recorded at 5 °C in H2O for the duplexes M9 and M10.

Figure S4. The H5-to-amine NOESY cross-peaks for cytidine residues for the duplex M10.

Figure S5 The imino proton regions in the NMR spectra recorded at 5 °C for M11-M12 and M13-M14.

Figure S6 The imino region of the NMR spectra as a function of temperature (NMR melting) for M19 and M20.

Table S1 The ΔCS induced by the G to s^6^G substitution for the M9-M10 duplex pair.

Table S2 The ΔCS induced by the G to s^6^G substitution for the M11-M12 duplex pair

**Schematic S1.** Chemical synthesis of protected 6-thioguanosine amidite. a = Ac_2_O, Et_3_N, DMAP, ACN; b = i-BuCl, Py; c = Lawesson’s reagent, dioxan; d = MeOH/NH_4_OH; e = DMTCl, Py; f = DNFB, Et_3_N, ACN; g = TBDMSCl, Py, imidazol; h= [(CH_3_)_2_CH]_2_NP(Cl)OCH_2_CH_2_CN, ACN, tetrazol

| 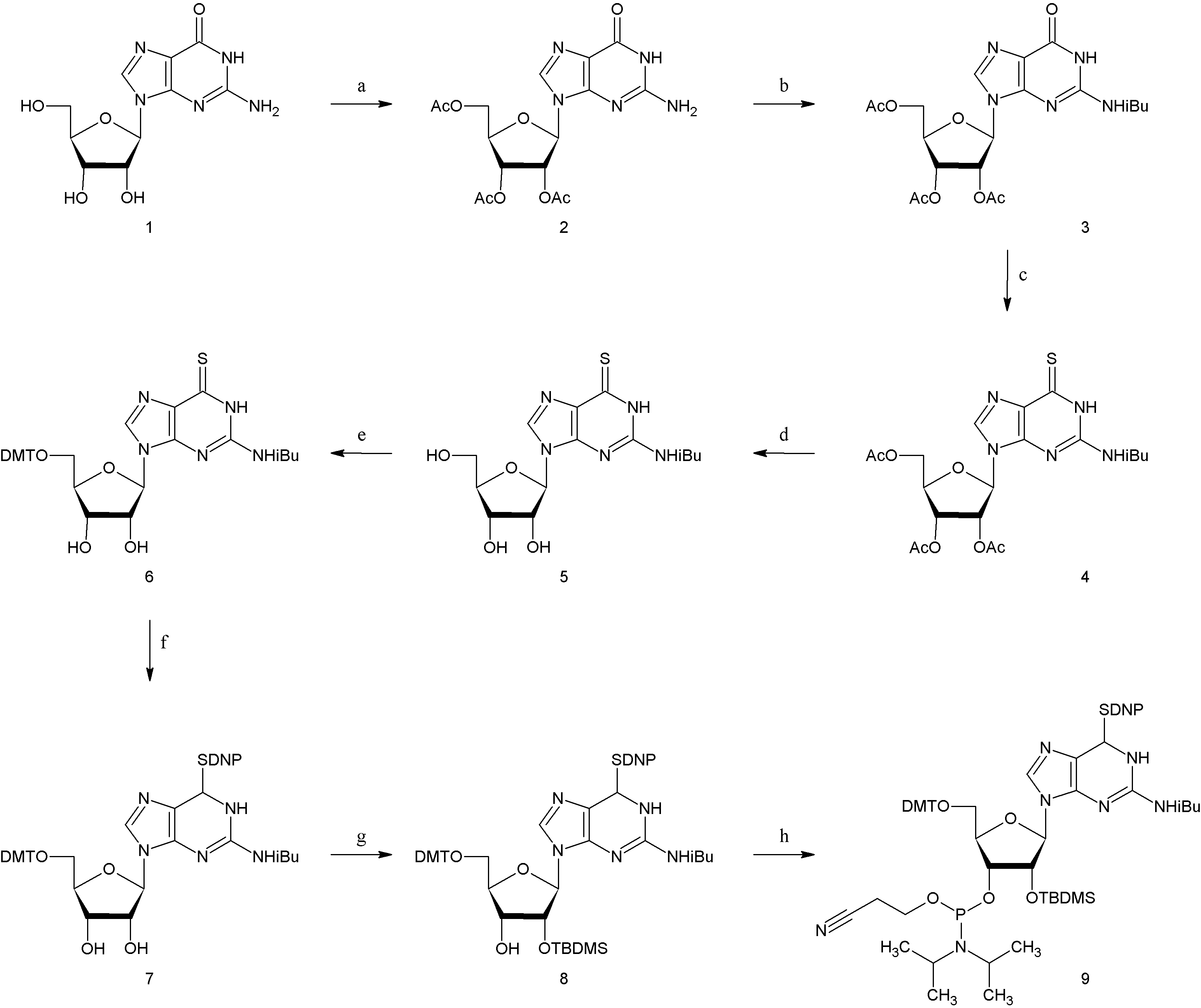 |
| --- |

**Synthesis of 2',3',5'-tri-O-acetyl-guanosine (1)**

Guanosine (8.5 g, 30 mmol) was suspended in 300 mL of acetonitrile, and DMAP (250 mg, 2.25 mmol) and 17 mL (118 mmol) of triethylamine were added. Acetic anhydride (10.2 ml, 108 mmol) was added to that mixture, and the reaction was incubated at room temperature for approximately 1 hour (until all reagents dissolved). Progress of the reaction was monitored by TLC (dichloromethane/methanol = 9/1 v/v). After the reaction was completed, acetonitrile was evaporated. The residue was dissolved in methylene chloride, and the organic layer was washed twice with a saturated aqueous solution of NaHCO_3_ and once with an aqueous solution of Na_2_HPO_4_. The organic layer was dried over anhydrous Na_2_SO_4_, filtered and evaporated to obtain 11.73 g of product (95% yield).

**^1^H NMR** (DMSO-d_6_) δ ppm: 2.04 (s, 3H, Ac), 2.05 (s, 3H, Ac), 2.11 (s, 3H, Ac), 6.00 (d, J=6.0 Hz, 1H, H1') and 7.94 (s, 1H, H8)

**Synthesis of 2',3',5'-tri-O-acetyl-N2-isobutyrylguanosine (2)**

Compound **1** (11.73 g, 28.65 mmol) was dissolved in 150 mL of pyridine, and isobutyryl chloride (6 mL 57.3 mmol) was added slowly. The reaction was conducted at room temperature for 12 hours. Progress of the reaction was monitored by TLC (dichloromethane/methanol = 95/5 v/v). After the substrate disappeared, the reaction mixture was slowly poured into a saturated aqueous solution of NaHCO_3_ and extracted three times with dichloromethane. The organic layer was dried over anhydrous Na_2_SO_4_, filtered and evaporated to obtain 12.12 g (88% yield) of crude product.

**^1^H NMR** (DMSO-d_6_) δ ppm: 1.11 (s, 3H, iBu), 1.13 (s, 3H, iBu), 2.03 (s, 3H, Ac), 2.05 (s, 3H, Ac), 2.12 (s, 3H, Ac), 6.08 (d, J=6.5 Hz, 1H, H1') 8.25 (s, 1H, H8); **^13^H NMR** (DMSO-d_6_) δ ppm: 154 (C6O guanosine)

**Synthesis of 2',3',5'-tri-O-acetyl-N2-isobutyryl-6-thioguanosine (3)**

Compound **2** (12.12 g, 26.7 mmol) was dissolved in 100 mL of dioxan, and Lawesson’s reagent (6.49 g, 16 mmol) was added. The reaction was conducted under reflux at 100 °C for approximately 2 hours. Progress of the reaction was monitored by TLC (dichloromethane/methanol = 95/5 v/v). After the substrate disappeared, the reaction mixture was cooled to room temperature. Then, the reaction mixture was slowly poured into a saturated aqueous solution of NaHCO_3_ and extracted three times with dichloromethane. The organic layer was dried over anhydrous Na_2_SO_4_, filtered and evaporated. The residue was purified by column chromatography (up to 2% methanol in dichloromethane). The yield of compound **3** was 11.34 g (90%).

**^1^H NMR** (DMSO-d_6_) δ ppm: 1.14 (s, 3H, iBu), 1.15 (s, 3H, iBu), 2.03 (s, 3H, Ac), 2.05 (s, 3H, Ac), 2.12 (s, 3H, Ac), 6.08 (d, J=6.5 Hz, 1H, H1') and 8.45 (s, 1H, H8); **^13^H NMR** (DMSO-d_6_) δ ppm: 174 (C6S guanosine)

**Synthesis of N2-isobutyryl-6-thioguanosine (4) and 5'-O-dimethoxytrityl-N2-isobutyryl-6-thioguanosine (5)**

Compound **3** (11.34 g, 22.7 mmol) was dissolved in a mixture of 60 mL of 25% aqueous ammonia and 60 mL of methanol. The reaction was carried out at room temperature for approximately 2 hours. Progress of the reaction was monitored by TLC (dichloromethane/methanol = 9/1 v/v). After the substrate disappeared, the reaction mixture was evaporated until dry. The yield of compound **4** was 8.5 g.

Compound **4** (8.5 g, approximately 22 mmol) was evaporated three times with pyridine and dissolved in 120 mL of dry pyridine. Dimethoxytrityl chloride (8.57 g, 25 mmol) was added. The reaction was carried out at room temperature for 12 hours. Progress of the reaction was monitored by TLC (dichloromethane/methanol = 95/5 v/v). After the substrate disappeared, the reaction mixture was poured into a saturated aqueous solution of NaHCO_3_ and extracted three times with dichloromethane. The organic layer was dried over anhydrous Na_2_SO_4_, filtered and evaporated. The residue was purified by silica gel column chromatography (up to 2% methanol in dichloromethane). The yield of compound **5** was 8.64 g (56%).

**^1^H NMR** (DMSO-d_6_) δ ppm: 1.13 (s, 3H, iBu), 1.15 (s, 3H, iBu), 3.72 (s, 6H, MeO-DMT), 65.87 (d, J=4.4 Hz, 1H, H1'), 6.8-7.3 (m, 13H, DMT) and 8.29 (s, 1H, H8)

**Synthesis of 5'-O-dimethoxytrityl-N2-isobutyryl-6S-dinitrophenyl-6-thioguanosine (6)**

Compound **5** (8.64 g, 12.90 mmol) was dissolved in 100 mL of acetonitrile, and 2.15 mL of trimethylamine (2.15 mmol) was added. Next, 1.78 mL of 1-fluoro-2,4-dinitrobenzene (2.64 g, 14.20 mmol) was added to the reaction mixture. The reaction was carried out at room temperature for approximately 2 hours. Progress of the reaction was monitored by TLC (dichloromethane/methanol = 9/1 v/v). After the substrate disappeared, the reaction mixture was evaporated until dry. The residue was dissolved in dichloromethane, and the organic layer was washed three times with an aqueous solution of NaHCO_3_. The organic layer was dried over anhydrous Na_2_SO_4_, filtered and evaporated to yield compound **6** (9.72 g, 90%).

**^1^H NMR** (DMSO-d_6_) δ ppm: 0.77 (t, J=5.2, 6H, iBu), 3.69 (s, 6H, MeO-DMT), 5.98 (d, J=4.4 Hz, 1H, H1'), 6.7-7.3 (m, 13H, DMT), 8.09 (d, J=8.9 Hz, 1H, H-6, DNP), 8.27 (d, J=8.9 Hz, 1H, H-5, DNP), 8.56 (s, 1H, H8) and 8.91 (d, J=2.5 Hz, 1H, H-3, DNP)

**Synthesis of 2’-*tert*-butyldimethylsilyl-5'-O-dimethoxytrityl-N2-isobutyryl-6S-dinitrophenyl-6-thioguanosine (7a)**

Compound **6** (9.72 g, 11.57 mmol) was evaporated twice with pyridine and dissolved in 60 mL of dry pyridine. Imidazole (2.05 g, 30 mmol) and *tert*-butyldimethylsilyl chloride (2.27 g, 15 mmol) were added. The reaction was carried out at room temperature for approximately 4 hours. Progress of the reaction was monitored by TLC (dichloromethane/methanol = 98/2 v/v). Afterwards, the substrate almost completely disappeared, and traces of disilyl-substituted product were visible. The reaction mixture was poured into a saturated aqueous solution of NaHCO_3_ and extracted three times with methylene chloride. The organic layer was dried over anhydrous Na_2_SO_4_, filtered and evaporated. The residue was purified by column chromatography (cyclohexane/chloroform = 25/75 v/v) yielding 2’-(**7a**) and 3’-(**7b**) isomers at approximate yields of 50% each.

Compound **7b** was dissolved in methanol and stirred for 24 hours at room temperature. Afterwards, TLC showed partial isomerization to product **7a**. The reaction mixture was evaporated until dry. The residue was purified by column chromatography as previously described. The isomerization procedure was repeated three times. Combined fractions of product **7a** from all reactions were lyophilized from benzene and stored at -20 °C.

**^1^H NMR** (DMSO-d_6_) δ ppm: -0.1 (s, 3H, Me-TBDMSi), 0.0 (s, 3H, Me-Si), 0.77 (s, 9H, tBu-TBDMSi), 1.0 (t, J=7.1, 6H, iBu), 3.70 (s, 6H, OMe-DMTr), 5.98 (d, J=4.1 Hz, 1H, H1'), 6.7-7.4 (m, 13H, DMTr), 8.13 (d, J=8.9 Hz, 1H, H-6, DNP), 8.35 (dd, J=8.9 Hz, 1H, H-5, DNP), 8.56 (s, 1H, H8) and 8.91 (d, J=2.5 Hz, 1H, H-3, DNP).

**Synthesis of 2’-*tert*-butyldimethylsilyl-3′-[2-cyanoethyl N,N-diisopropylphosphoramidite]-5'-O-dimethoxytrityl-N2-isobutyryl-6S-dinitrophenyl-6-thioguanosine (8)**

Compound **7a** (1.43 g, 1.5 mmol) and tetrazole (0.105 g, 1.5 mmol) in a 50 mL round bottom flask were dried under high-vacuum for 3 hours. Afterwards, 8 mL of dry acetonitrile was added with a syringe. The mixture was stirred at room temperature until tetrazole had dissolved, and 2-cyanoethyl N,N,N′,N′-tetraisopropylphosphorodiamidite (0.6 g, 1.95 mmol) was then added. The reaction was carried out at room temperature for 12 hours. Progress of the reaction was monitored by reversed-phase TLC (acetone/water = 8/2 v/v). After the substrate disappeared, the reaction mixture was diluted with dichloromethane containing 2% triethylamine and poured into a saturated aqueous solution of NaHCO_3_. The product was extracted three times with dichloromethane containing 2% triethylamine. The organic layer was dried over anhydrous Na_2_SO_4_, filtered and evaporated. The residue was purified by silica gel column chromatography with the addition of 2% triethylamine (hexane/ethyl acetate, up to 100% ethyl acetate) to obtain compound **8**. The product was lyophilized from benzene. The yield of the product was 1.2 g (56%).

**^1^H NMR** (AcCN-d_3_) δ ppm: 0.85 (s, 9H, tBu-TBDMSi), 0.95-1.05 (m, 6H, iBu), 1.15-1.20 (m, 12H, iPr-N), 3.74 (s, 6H, OMe-DMTr), 5.97 (2xd, J=6.3 Hz, 1H, H1'), 6.7-7.5 (m, 13H, DMTr), 8.13 (d, J=8.9 Hz, 1H, H-6, DNP), 8.23 (s, 1H, H8), 8.27 (dd, J=8.9 Hz, 1H, H-5, DNP) and 8.88 (d, J=2.5 Hz, 1H, H-3, DNP); **^31^P NMR** (AcCN-d_3_) δ ppm: 150.18 and 148.69

**NMR assignments of RNA duplexes**

The nonexchangeable aromatic and imino protons were assigned in the spectra recorded in 100 % D_2_O, using the standard “NOESY-walk” (Figure S2). The amino protons of cytidines were then identified in the NOESY spectra recorded in H_2_O by their very strong cross-peaks to the aromatic H5 atoms of the given cytidine (Figure S3). Finally, the imino protons were assigned based on the very strong A-H2 to U-imino cross-peaks (for A-U pairs) or C-NH_2_ to G-imino cross-peaks (for G-C pairs) and confirmed based on the imino-imino connectivities (Figure S4).

Figure S1 The CD spectra recorded for the duplex pairs: (a) M9-M10, (b) M11-M12, (c) M13-M14, (d) M15-M16, (e) M17-M18, (f) M19-M20, (g) M21-M22

| a) | 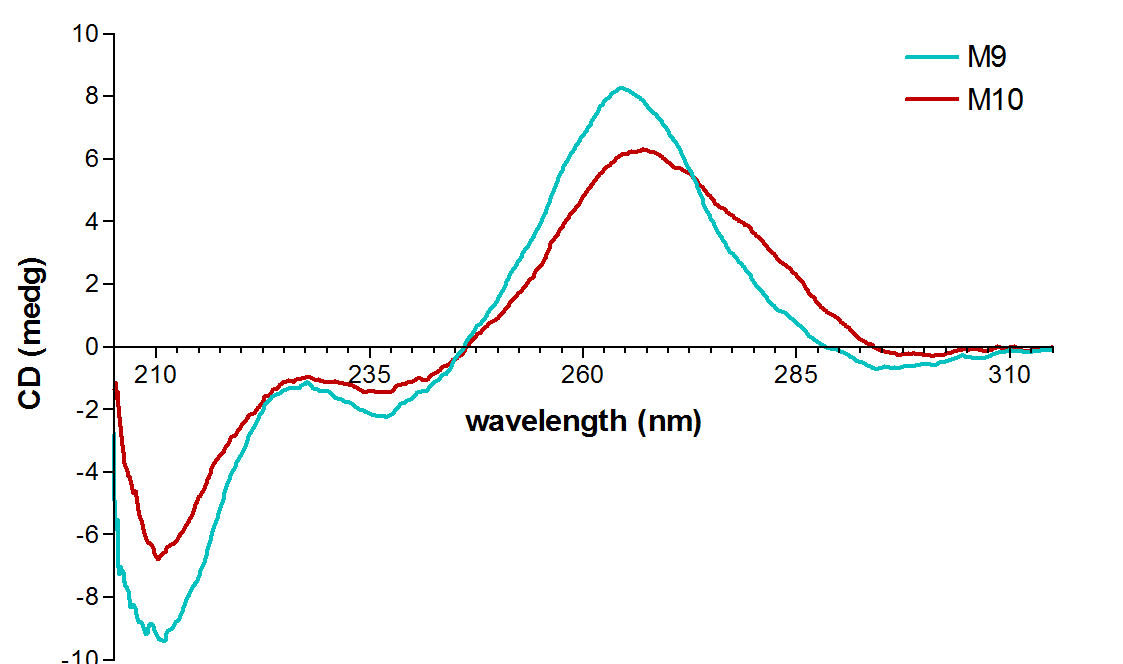 |
| --- | --- |
| b) | 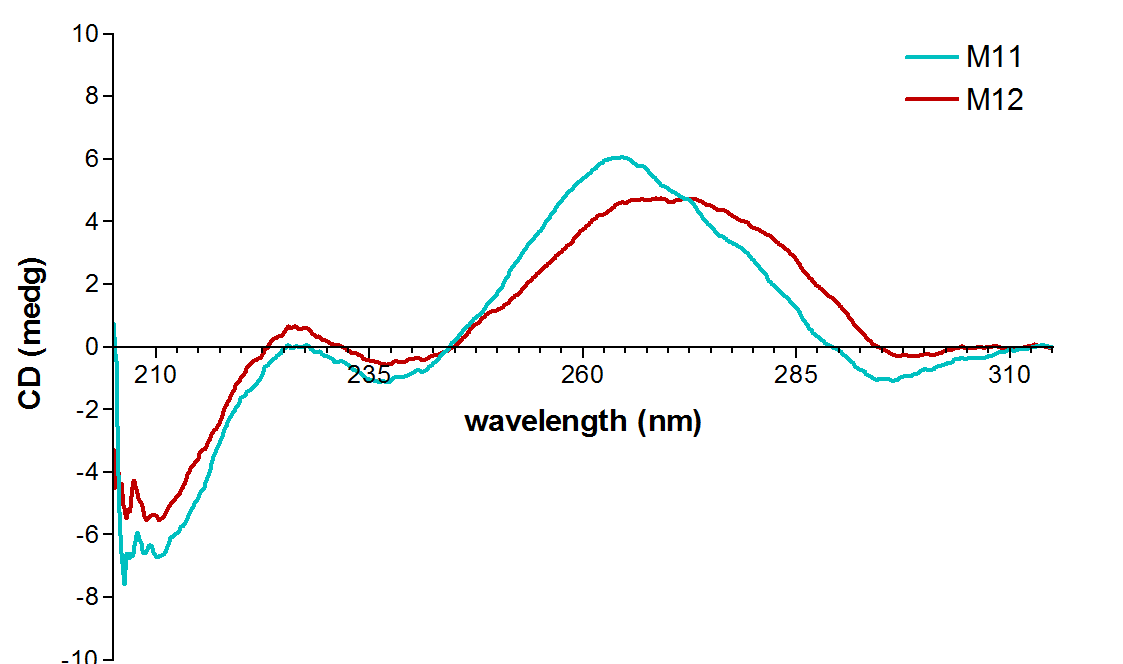 |
| c) | 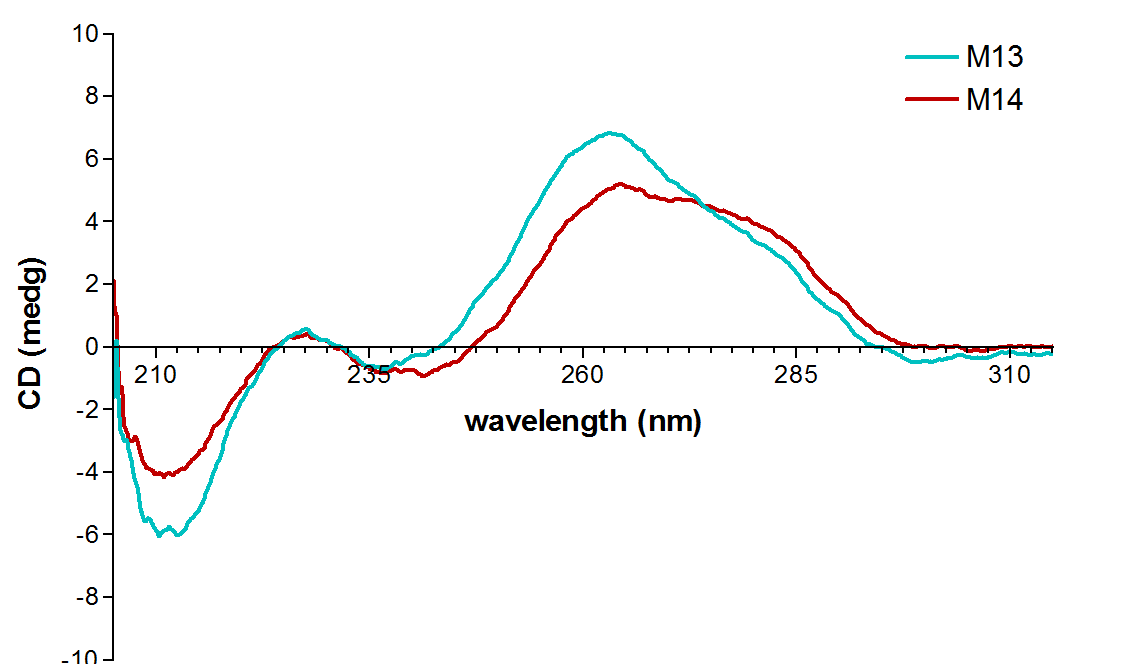 |
| d) | 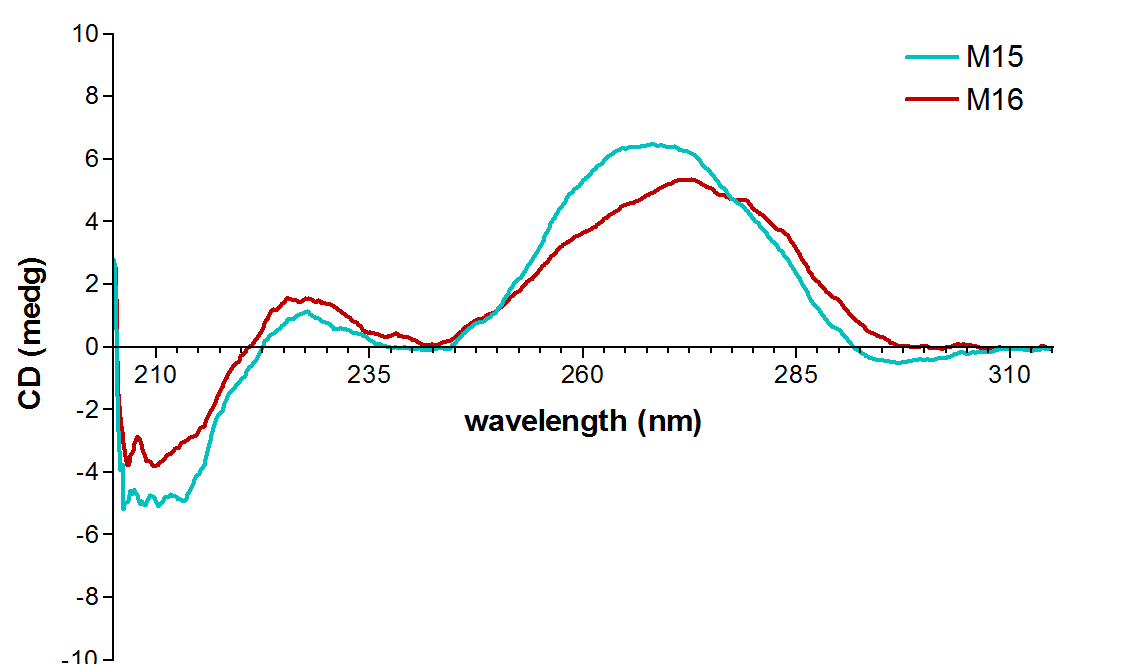 |
| e) | 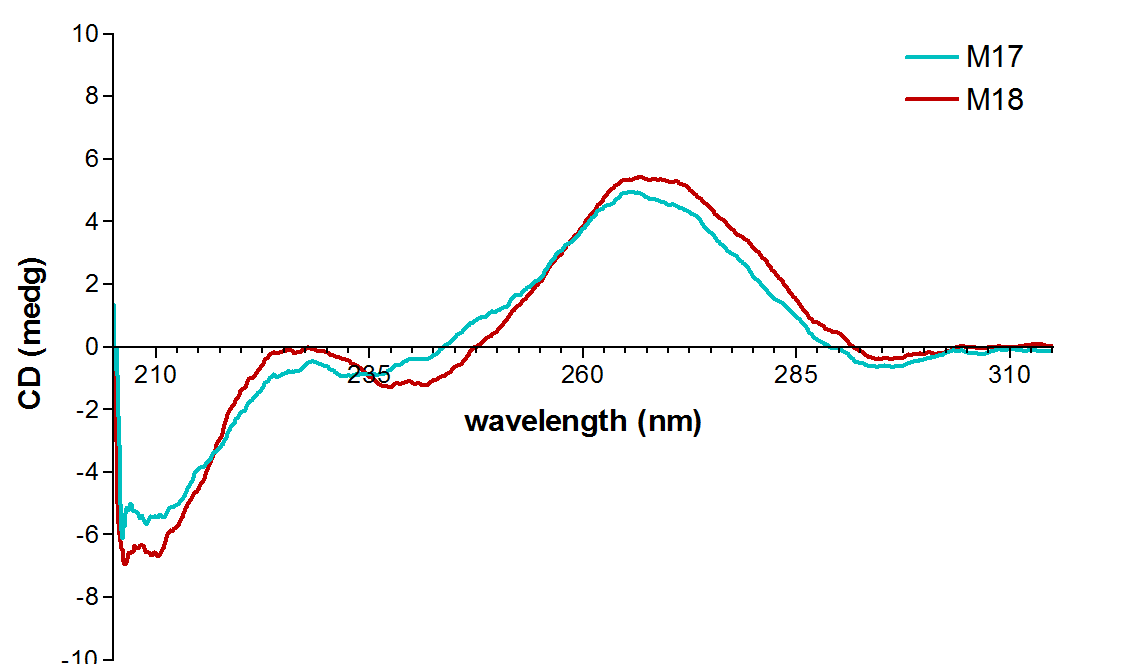 |
| f) | 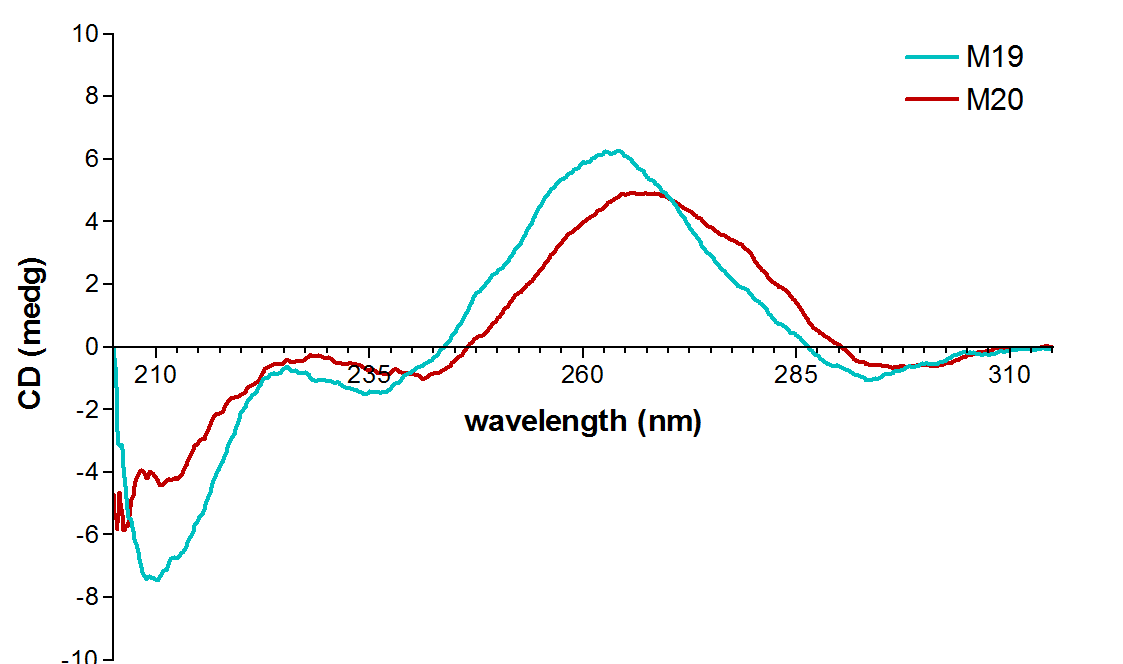 |
| g) | 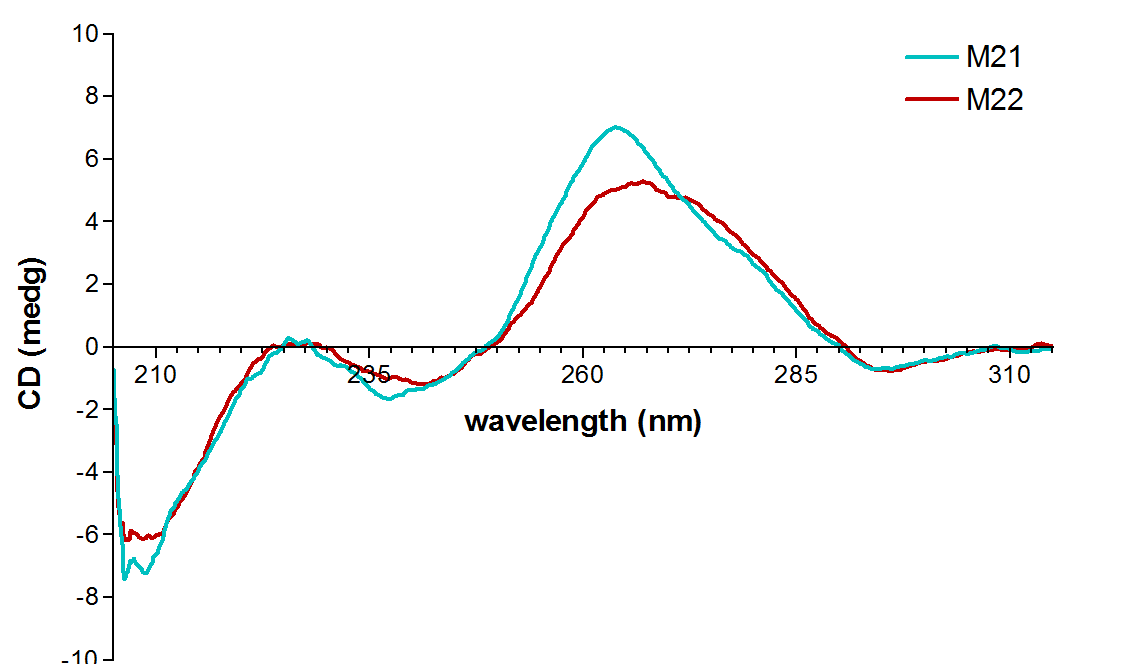 |

Figure S2. The aromatic-to-anomeric section of the NOESY spectra recorded at 25 °C in D_2_O for the duplexes M9 (left) and M10 (right). The “NOESY-walks” used to assign the resonances are marked in each panel with blue (strand 1; residues 1 to 9) and green lines (strand 2; residues 10 to 18).

| 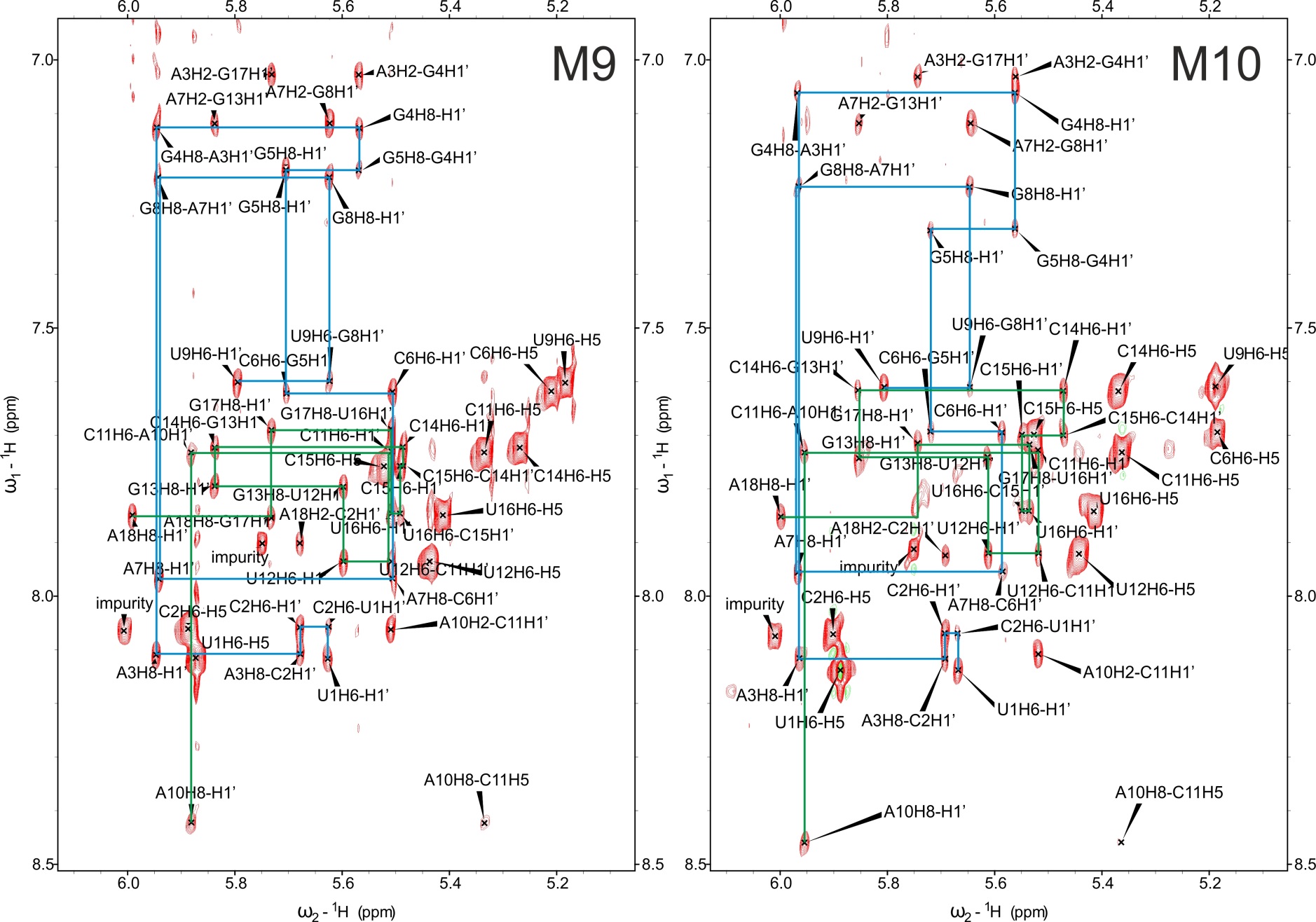 |
| --- |

Figure S3. The H5-to-amine cross-peaks for cytidine residues in the NOESY spectrum recorded at 5 °C in H_2_O for the duplex M10.

| 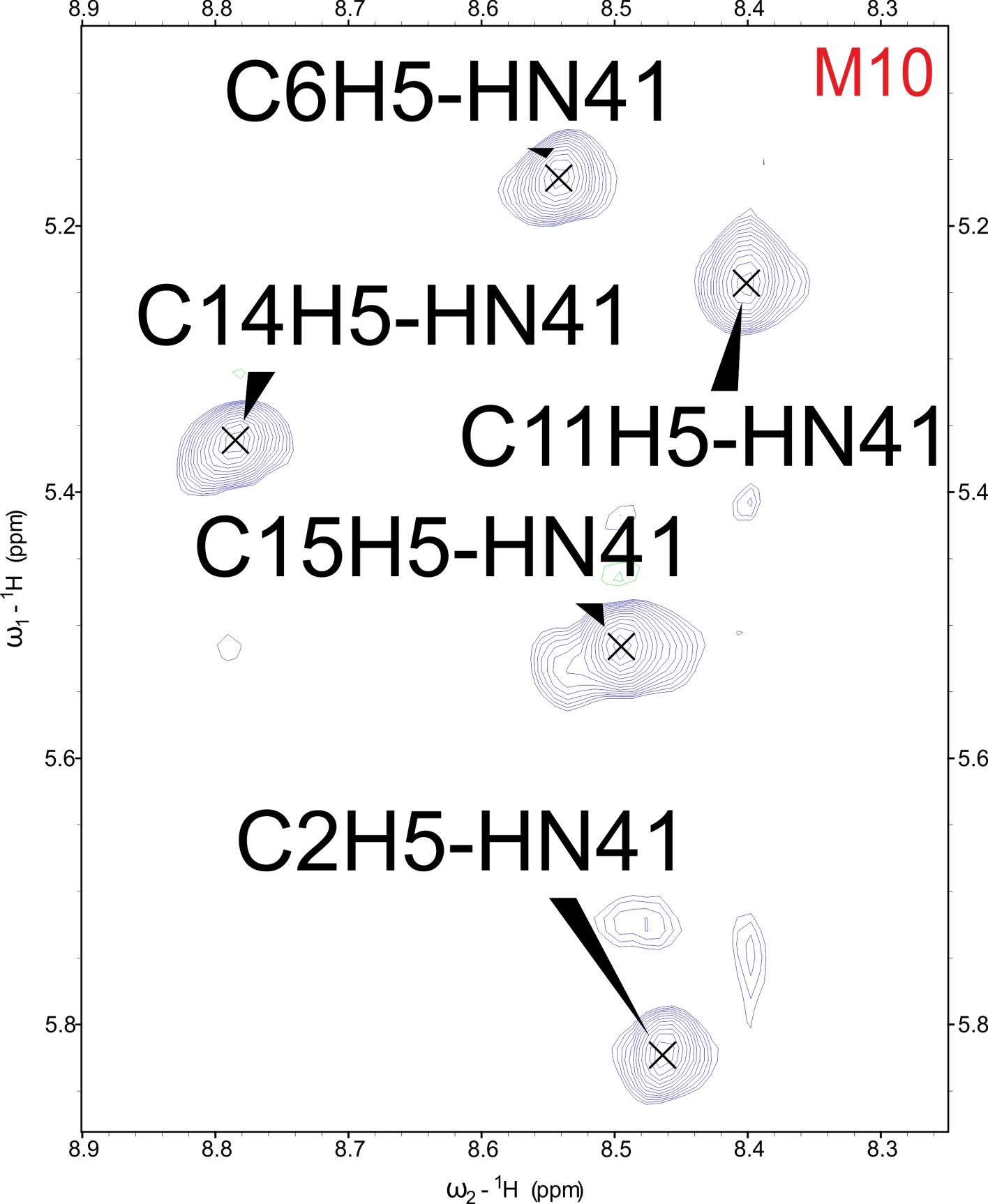 |
| --- |

Figure S4. The imino-imino and imino-amino sections of the NOESY spectra recorded at 5 °C in H_2_O for the duplexes M9 (top) and M10 (bottom). Only the cross-peaks essential to the assignment of imino protons are marked (see Supplementary Methods).

| 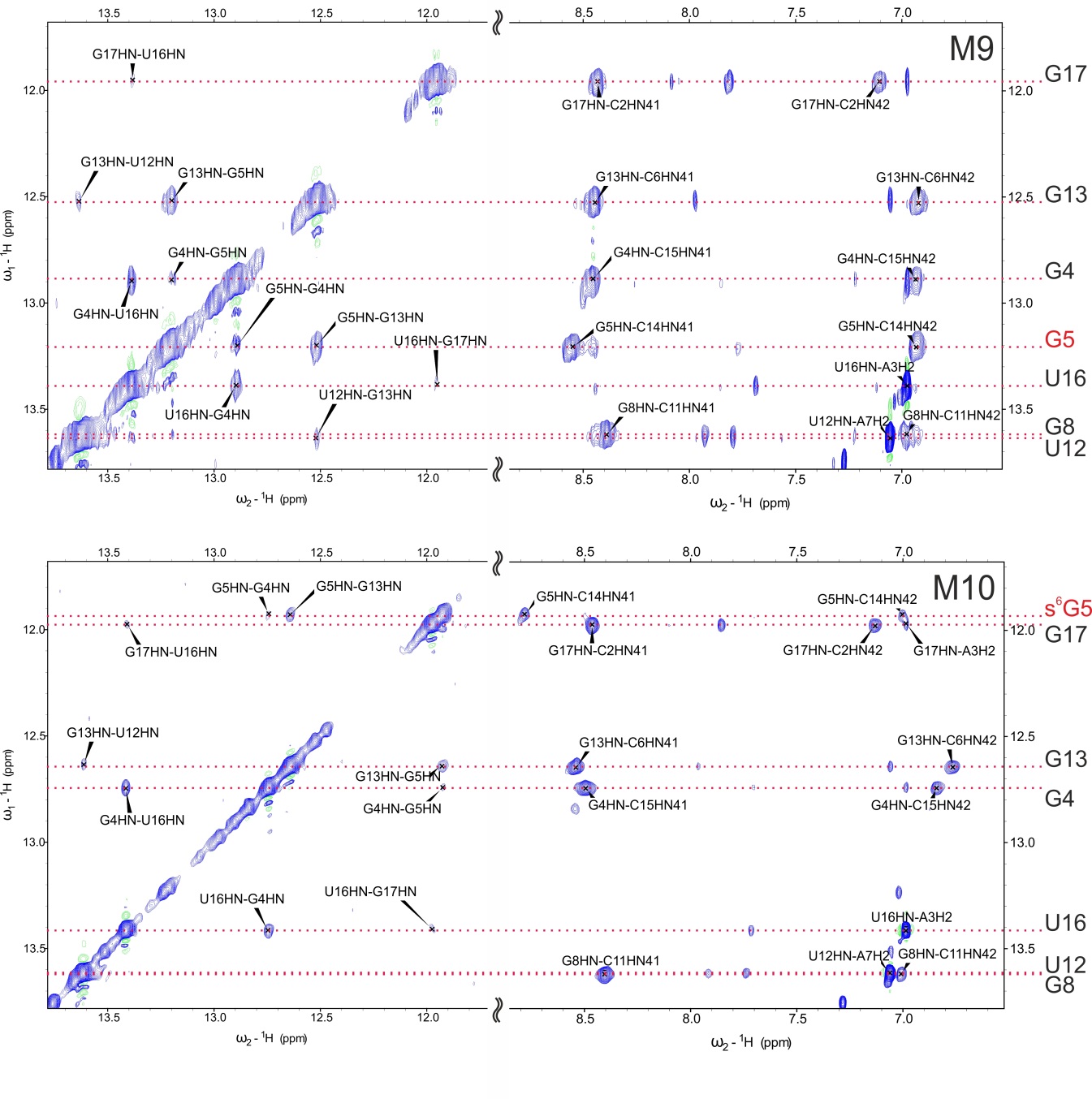 |
| --- |

Figure S5 The imino proton regions in the NMR spectra recorded at 5 °C for (a) the M11-M12 and (b) the M13-M14 duplex pairs. The resonances due to the modified nucleotide G5/s6G5 are marked in red.

| 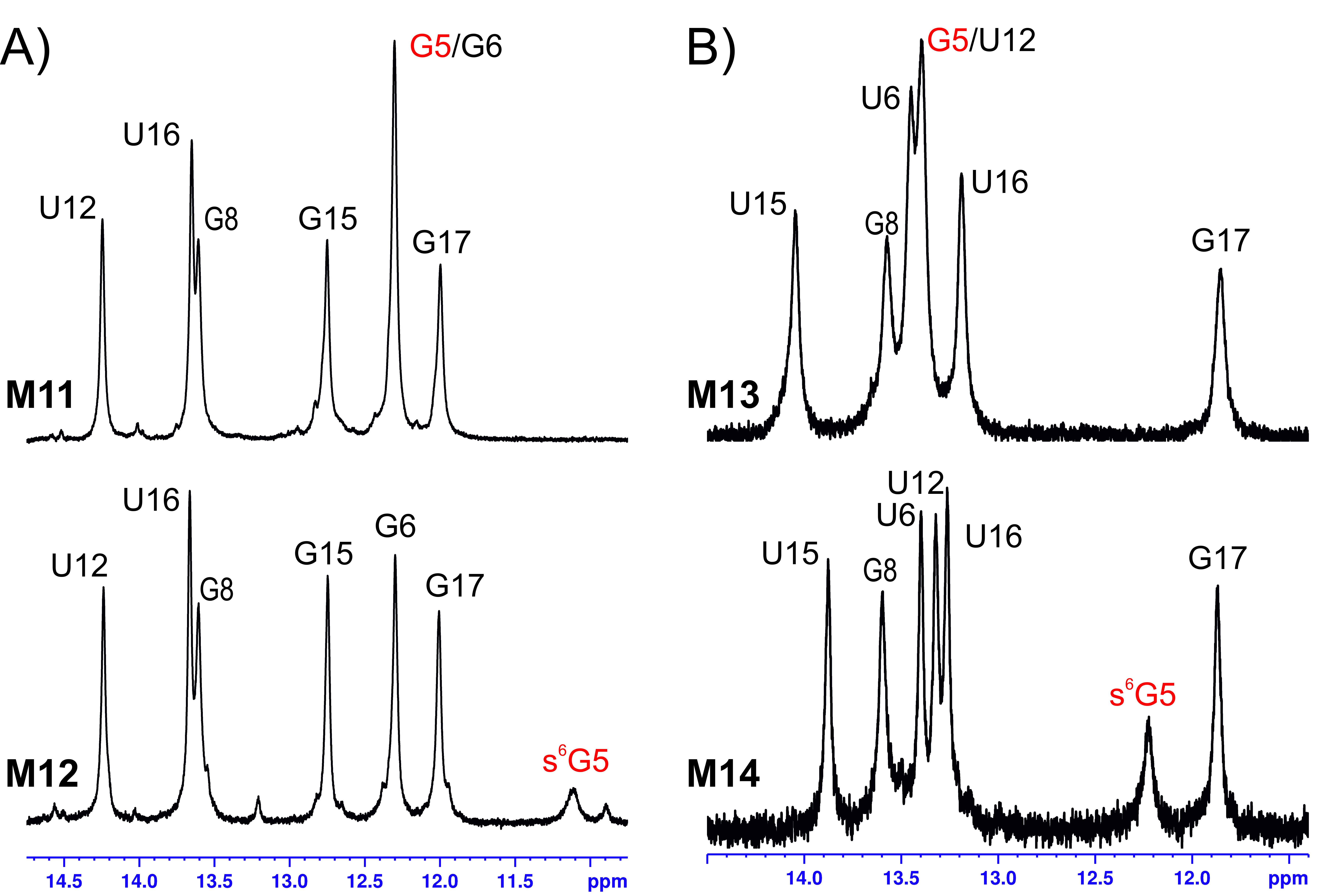 |
| --- |

Figure S6 The imino region of the NMR spectra as a function of temperature (NMR melting) for (a) M20 and (b) M19.

| 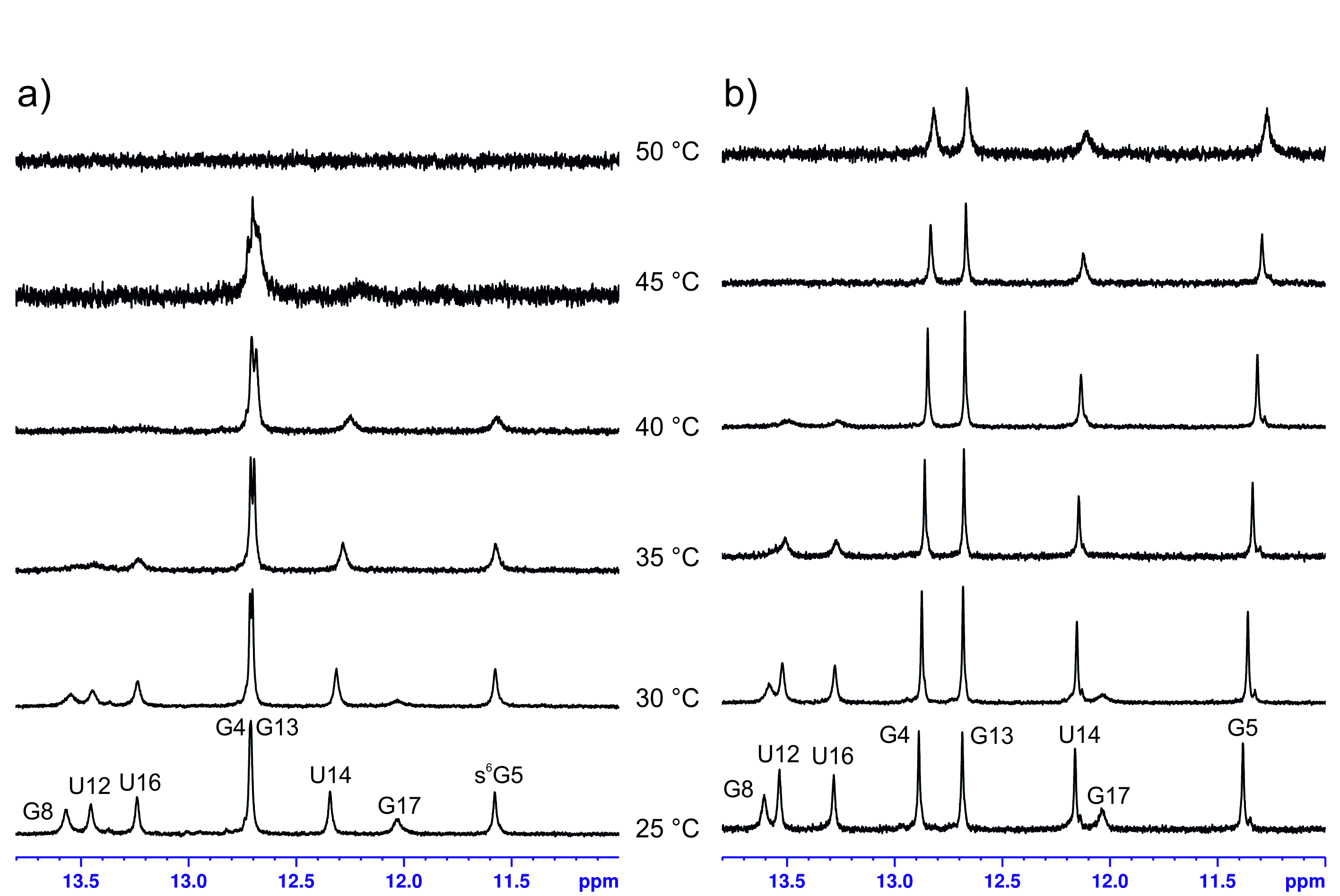 |
| --- |

Table S1. The chemical shift changes (ΔCS) induced by the G to s^6^G substitution for the M9-M10 duplex pair. The ΔCS greater than 0.05 ppm are given in bold.

| strand 1  residue | strand 1  nonexchangeable protons | | | exchangeable protons | | | strand 2  nonexchangeable protons | | | strand 2  residue |
| --- | --- | --- | --- | --- | --- | --- | --- | --- | --- | --- |
|  | H1’ | H8/H6 | H2/H5 | imino^a^ | amino^b^  (bonding) | amino^b^  (free) | H2/H5 | H8/H6 | H1’ |  |
| U1 | 0.042 | 0.022 | 0.015 | --- | na | na | 0.024 | 0.003 | 0.007 | A18 |
| C2 | 0.014 | 0.010 | 0.014 | 0.018 | 0.032 | 0.033 | na | 0.028 | 0.012 | G17 |
| A3 | 0.020 | 0.008 | 0.004 | 0.024 | na | na | 0.003 | 0.006 | 0.028 | U16 |
| G4 | 0.008 | **0.065** | na | **0.15** | **0.096** | 0.042 | 0.005 | **0.058** | **0.057** | C15 |
| G5 | 0.016 | **0.111** | na | **1.277** | **0.238** | **0.070** | **0.100** | **0.105** | 0.014 | C14 |
| C6 | **0.079** | **0.074** | 0.025 | **0.122** | **0.152** | **0.098** | na | **0.052** | 0.016 | G13 |
| A7 | 0.025 | 0.011 | 0.001 | 0.023 | na | na | 0.006 | 0.014 | 0.014 | U12 |
| G8 | 0.023 | 0.017 | na | 0.003 | 0.013 | 0.037 | 0.029 | 0.001 | 0.009 | C11 |
| U9 | 0.011 | 0.008 | 0.004 | --- | na | na | 0.047 | 0.036 | **0.074** | A10 |

^a^ The imino proton of guanosine (GC pairs) or uridine (AU pairs) of the given base pair

^b^ The amino protons of cytidine of the given base pair

Table S2. The chemical shift changes (ΔCS) induced by the G to s^6^G substitution for the M11-M12 duplex pair. The ΔCS greater than 0.05 ppm are given in bold.

| strand 1  residue | strand 1  nonexchangeable protons | | | exchangeable protons | | | strand 2  nonexchangeable protons | | | strand 2  residue |
| --- | --- | --- | --- | --- | --- | --- | --- | --- | --- | --- |
|  | H1’ | H8/H6 | H2/H5 | imino^a^ | amino^b^  (bonding) | amino^b^  (free) | H2/H5 | H8/H6 | H1’ |  |
| U1 | 0.013 | 0.010 | 0.003 | --- | na | na | 0.001 | 0.017 | 0.011 | A18 |
| C2 | 0.005 | 0.014 | 0.001 | 0.009 | 0.028 | 0.006 | na | 0.019 | 0.015 | G17 |
| A3 | 0.007 | 0.009 | 0.014 | 0.007 | na | na | 0.008 | 0.014 | 0.033 | U16 |
| C4 | 0.012 | 0.012 | 0.002 | 0.006 | **0.062** | **0.112** | na | 0.023 | 0.043 | G15 |
| G5 | 0.015 | **0.122** | na | --- | **0.377** | **0.160** | 0.024 | 0.012 | **0.051** | C14 |
| G6 | **0.062** | **0.109** | na | 0.005 | 0.004 | 0.016 | 0.020 | 0.028 | 0.001 | C13 |
| A7 | 0.016 | 0.010 | 0.006 | 0.013 | na | na | 0.001 | 0.011 | 0.006 | U12 |
| G8 | 0.012 | 0.014 | na | 0.003 | 0.010 | 0.008 | 0.009 | 0.01 | 0.002 | C11 |
| U9 | 0.011 | 0.019 | 0.005 | --- | na | na | 0.018 | 0.011 | 0.029 | A10 |

^a^ The imino proton of guanosine (GC pairs) or uridine (AU pairs) of the given base pair

^b^ The amino protons of cytidine of the given base pair
